# Association of cumulative social risk with mortality and adverse cardiovascular disease outcomes

**DOI:** 10.1186/s12872-017-0539-9

**Published:** 2017-05-08

**Authors:** Sebhat Erqou, Justin B. Echouffo-Tcheugui, Kevin E. Kip, Aryan Aiyer, Steven E. Reis

**Affiliations:** 1 Department of Medicine, University of Pittsburgh, Pittsburgh, PA USA; 2 Department of Medicine, Brigham Women’s Hospital, Boston, MA USA; 3 College of Nursing, University of South Florida, Tampa, FL USA

**Keywords:** Social risk factors, Cumulative social risk, Cardiovascular disease, Racial disparity

## Abstract

**Background:**

Quantifying the cumulative effect of social risk factors on cardiovascular disease (CVD) risk can help to better understand the sources of disparities in health outcomes.

**Method and results:**

Data from the Heart Strategies Concentrating on Risk Evaluation (HeartSCORE) study were used to create an index of cumulative social risk (CSR) and quantify its association with incident CVD and all-cause mortality. CSR was defined by assigning a score of 1 for the presence of each of 4 social factors: i) racial minority status (Black race), ii) single living status, iii) low income, and iv) low educational level. Hazard ratios (HRs) were computed using Cox-regression models, adjusted for CVD risk factors. Over a median follow-up period of 8.3 years, 127 incident events were observed. The incidence of the primary outcome for subgroups of participants with 0, 1, and ≥2 CSR scores was 5.31 (95% CI, 3.40–7.22), 10.32 (7.16–13.49) and 17.80 (12.94–22.67) per 1000 person-years, respectively. Individuals with CSR score of 1 had an adjusted HR of 1.85 (1.15–2.97) for incident primary outcomes, compared to those with score of 0. The corresponding HR for individuals with CSR score of 2 or more was 2.58 (1.60–4.17).

**Conclusion:**

An accumulation of social risk factors independently increased the likelihood of CVD events and deaths in a cohort of White and Black individuals.

**Electronic supplementary material:**

The online version of this article (doi:10.1186/s12872-017-0539-9) contains supplementary material, which is available to authorized users.

## Background

Racial disparities in cardiovascular disease (CVD) risk and mortality is an important challenge for health care in the United States and worldwide [1–4]. The Eight Americas Study indicated that CVD represents the main driver of disparities in life expectancy across various racial/ethnic groups in the United States [5]. We have previously reported that Black race is associated with CVD risk independent of several novel and traditional CVD risk factors [6]. While studies of biological determinants of such disparities are important, current evidence suggests that racial differences in CVD incidence are mainly related to social and environmental factors, such as socioeconomic deprivation (e.g., low family income, low education level). Myriad, complex and interacting downstream social and psychological constructs such as access to healthcare, medical compliance, eating habits, depression and stress are thought to play role in this relationship [7–11].

Socially disadvantaged individuals often have multiple types of social risk factors. However, few studies to date have investigated the cumulative effect of multiple social risk factors on CVD risk [7, 8]. Identification of the cumulative effect of various social risk factors on CVD risk can provide insight into the real-world effects of social disadvantage on CVD and may inform approaches to addressing CVD disparities that extend beyond strategies focusing on a single risk factor. For example, a recent study of NHANES III data reported the cumulative effect of four social risk factors (i.e., belonging to an ethnic minority group, low income, low education, single living status) on risk of CVD mortality and all-cause mortality [7]. Each additional social risk score was found to be associated with a 17% higher risk of CVD mortality and 43% higher risk of under 65-year old mortality [7]. However, the NHANES report did not include non-fatal CVD events in the outcome. In order to further understand the role of socio-environmental factors in CVD disparities, we used data from the Heart Strategies Concentrating on Risk Evaluation (HeartSCORE) study to create an index of cumulative social risk (CSR) and quantify its association with incident CVD events and all-cause mortality. We also assessed the cross-sectional association between CSR and carotid intima-media thickness (CIMT), which is a non-invasive intermediate marker of atherosclerosis.

## Methods

### Study population

HeartSCORE is an ongoing community-based prospective cohort study of racial disparities of CVD comprised of 2000 participants with approximately equal representation of Blacks (44%) and Whites (56%). Fifty-one participants (2.6%) were identified to be of other minority group (other than Black) and were excluded from the present paper, as data were too small for meaningful analyses. The methods of HeartSCORE have been described previously [12, 13]. Eligibility criteria included age 45 to 75 years at study entry, residence in the greater Pittsburgh metropolitan area, ability to undergo baseline and annual follow-up visits, and absence of known co-morbidities expected to limit life expectancy to less than 5 years.

### Social risk factors assessment

Demographic and medical histories were collected at the baseline visit. Race was self-reported. Participants completed a detailed questionnaire about their marital/cohabitating status, maximum education level achieved, annual income and ability to pay for basic needs. Single living status was defined as individuals who are not married or not cohabitating with a partner. Low income was defined as individuals with an annual income of less than $20 K or those reporting difficulty paying for their basic needs. Low educational level was defined as those who did not complete a high school diploma.

### Cumulative social risk

The components of CSR were defined by assigning a score of 1 for the presence (0 in their absence) of the following 4 social factors: i) racial minority status (Black race), ii) low income and, iii) low educational level, and iv) single living status. The individual scores were summed to give the CSR score (range 0 to 4). Participants with a score of 2, 3 or 4 were combined into one category for analyses due to small numbers in each of these categories. Subsidiary analyses were performed by combining only participants with score of 3 or 4 into one category, while leaving the rest as separate categories.

### Outcomes

Participants were assessed for incident hospitalization and CVD events by semi-annual questionnaires and during annual follow-up study visits. Incident CVD events were pre-defined as non-fatal myocardial infarction, acute coronary syndrome, stroke, coronary revascularization, or cardiac death. The primary outcome of interest was a composite of CVD events and all-cause mortality. We selected all-cause mortality as component of the primary outcome because social risk factors likely influence not only CVD risk but also global risk of mortality. The larger number of composite outcome events also allows for sufficient power to determine the association more precisely. A secondary outcome was composed of nonfatal and fatal CVD events. Events were confirmed and classified by reviews of medical records, including death certificates obtained from the Commonwealth of Pennsylvania. Additionally, we investigated the presence of significant subclinical atherosclerosis defined as maximal carotid artery intima-media thickness (CIMT) > 1 mm. Carotid artery imaging was carried out using a GE VIVID7 (General Electric Corp.) ultrasound imaging system and a 7 MHz linear array vascular ultrasound probe, using methods described previously [14].

### Covariates assessment

Depression, stress and perceived discrimination were assessed using validated questionnaires. Depression was assessed using the Center for Epidemiologic Studies Depression Scale (CES-D) [15] and the assessment of perceived stress was based on the Cohen Stress Scale [16].Perceived discrimination was assessed using a standard questionnaire that has been previously validated for population research [17]. The total scores were calculated by summing the “Yes” or “No” responses for the individual questions within the respective questionnaires. Depression was defined as CES-D score ≥ 16 [15].

Physical activity was assessed using the Lipid Research Clinic questionnaire [18], which includes questions about type and frequency of physical activity at work and during leisure time and permits classification of individuals as very active, moderately active, and inactive. The questionnaire provided approximations of ideal physical activity as defined by the American Heart Association (AHA) Life’s Simple 7 (LS7) [19, 20]. Consumption of fruits and vegetables was assessed using PrimeScreen questionnaire [21]. This self-administered questionnaire evaluates diet quality using average frequency of consumption of specific foods and food groups during the previous year. A value of 3 servings/d of fruits and vegetables on the PrimeScreen questionnaire has been shown to correlate closely with 5 servings/d when derived from more extensive food frequency questionnaires [21]. The questionnaire was used to define ideal consumption of fruits and vegetables according to AHA LS7 [19, 20].

Physical examination included measurement of vital signs and anthropometric measures of body fat distribution. Body mass index (BMI) was calculated as weight/height^2^ (kg/m^2^). Diabetes mellitus was defined as fasting glucose >126 mg/dL (the definition for diabetes at the time of study initiation) or a history of previously diagnosed diabetes. Fasting blood glucose was measured using the glucose oxidase method. Measurement of high-sensitivity C-reactive protein (hsCRP) was performed using an immunoturbidimetric assay on the Roche P Modular system (Roche Diagnostics - Indianapolis, IN), using reagents and calibrators from DiaSorin (Stillwater, MN). Serum interleukin-6 (IL6) concentrations were measured using commercially available ELISA assay kits (R&D Systems, Minneapolis, MN). To confirm reproducibility as reported by the kit manufacturer, a random subset of samples (10%) was assayed in duplicate.

### Statistical methods

We first created an index of CSR score for each HeartSCORE participant. We then assessed factors associated with CSR including socio-demographic, biophysical and biochemical variables. Test parameters were derived from analyses of variance for continuous variables, and chi-squared tests for categorical variables.

Cox-regression models were fit to examine the associations of CSR with incident outcomes. Adjustment was made for traditional CVD risk factors (i.e., age, sex, smoking status, blood pressure, diabetes, BMI, total and high-density lipoprotein cholesterol [HDL-c]). Further adjustments were made for variables that could potentially mediate the putative association between CSR and CVD/mortality outcomes, i.e., inflammatory variables (hsCRP and IL6), psychological variables (depression, stress and perceived discrimination scores), and statin use. We also constructed an alternative model by adjusting for ideal physical activity, and fruit and vegetable consumption as defined by AHA, in addition to the basic model comprised of the traditional CVD risk factors.

The cross-sectional association of CSR with significant CIMT (i.e., CIMT > 1 mm) was assessed using logistic regression models, adjusting for traditional CVD risk factors. We conducted a sensitivity analysis to examine the effect of various ways of defining and categorizing the CSR score. All analyses were performed using Stata software (Stata Corp., version 11, Texas, USA). *P*-values <0.05 were considered statistically significant.

## Results

### Baseline characteristics and correlates of race and CSR

The baseline characteristics of participants and factors associated with CSR are shown in Table 1. In total, 1731 (out of 1949) participants with complete information on all variables were included in the analyses. Among these participants, 35%, 30% and 35% had CSR scores of 0, 1 and ≥2, respectively. The proportions of current smokers and individuals with diabetes, hypertension, or family history of coronary artery disease increased across categories of CSR from 0, 1, and ≥2. Mean systolic blood pressure, diastolic blood pressure and BMI, as well as hsCRP and IL6 concentrations increased across categories of CSR. Similarly, depression, stress and perceived discrimination scores increased across the categories of CSR (Table 1). Thus, the CSR index was univariately associated with a broad spectrum of CVD risk factors.

**Table 1:** Study characteristic by cumulative social risk (3 categories)

Traditional risk factors	Overall *N* = 1731	CSR = 0 *N* = 607	CSR = 1 *N* = 520	CSR ≥2 *N* = 604	*p*-value
Age (yrs)	59.1 (7.4)	59.7 (7.3)	59.2 (7.6)	58.3 (7.4)	0.004
Male	593 (34.3%)	270 (44.5%)	185 (35.6%)	138 (22.9%)	<0.001
Smoker	188 (10.9%)	43 (7.1%)	44 (8.5%)	101 (16.7%)	<0.001
SBP (mmHg)	136.2(19.4)	133.1 (18.1)	134.9 (18.2)	140.9 (20.9)	<0.001
DBP (mmHg)	80.7 (10.4)	79.0 (10.0)	80.4 (9.8)	82.6(10.9)	<0.001
Diabetes	169 (9.8%)	31 (5.1%)	43 (8.3%)	95 (15.7%)	<0.001
Hypertension	727 (42.0%)	190 (31.3%)	205 (39.4%)	332 (55.0%)	<0.001
BMI (kg/m^2^)	30.0 (6.1)	28.3 (5.3)	29.8 (5.8)	31.7 (6.8)	<0.001
TC (mg/dL)	213.2 (42.6)	214.3 (41.4)	213.6 (41.3)	211.9 (44.9)	0.613
HDL-c (mg/dL)	57.6 (14.8)	56.8 (15.2)	57.1(14.2)	58.9 (14.9)	0.029
TG (mg/dL)	122.6 (73.3)	130.8 (74.9)	122.6 (70.9)	114.3 (72.8)	<0.001
Components of CSR					
Race - Black	715 (41.3%)	0 (0.0%)	208 (40.0%)	507 (83.9%)	<0.001
Low income	500 (28.9%)	0 (0.0%)	104 (20.0%)	396 (65.6%)	<0.001
Single living	722 (41.7%)	0 (0.0%)	197 (37.9%)	525 (86.9%)	<0.001
< High school	38 (2.2%)	0 (0.0%)	11 (2.1%)	27 (4.5%)	<0.001
Other variables					
Statin	361 (20.9%)	141 (23.2%)	108 (20.8%)	112 (18.6%)	0.14
Family hx CAD	628 (36.3%)	194 (32.0%)	182 (35.0%)	252 (41.7%)	<0.001
Depression^a^	204 (11.8%)	39 (6.4%)	54 (10.4%)	111 (18.4%)	<0.001
CESD score	6.86 (8.0)	5.29 (6.26)	6.50 (7.66)	8.74 (9.30)	<0.001
Cohen score	4.26 (3.01)	3.71 (2.68)	4.28 (2.99)	4.78 (3.26)	<0.001
DIS score	11.23 (6.2%)	9.58 (6.03)	11.43 (6.10)	12.73 (5.90)	<0.001
Creatinine (mg/dl)	0.92 (0.27)	0.91 (0.18)	0.93 (0.33)	0.93 (0.29)	0.419
HsCRP (mg/l)	2.91 (4.98)	2.34 (4.62)	2.64 (3.65)	3.73 (6.10)	<0.001
IL6 (pg/ml)	2.21 (2.02)	1.78 (1.48)	2.20 (1.63)	2.66 (2.62)	<0.001

*CSR* cumulative social risk score, *Hx* history, *BP* blood pressure, *BMI* body mass index, < High school – did not complete high school, *TC* total cholesterol, *HDL-c* high-density lipoprotein cholesterol, *TG* triglycerides, *HsCRP* high-sensitivity C-reactive protein, *IL6* interleukin-6, *CESD* Center for Epidemiologic Studies Depression Scale, Cohen stress scale, *DIS* discrimination score. ^a^Depression was defined as CES-D score ≥ 16. NB. The *p*-value is a test for statistical significant difference in the distribution of the respective variables across the categories of CSR. A *p*-value <0.05 is considered statistically significant

Analyses of baseline characteristics by race showed that White participants were older than Black participants on average (mean age 60 vs 58 years), and were less likely to be current smokers (8% vs 14%), have diabetes (5% vs 16%), or have hypertension (31% vs 56%). Black participants were less likely to be male (30% vs 37%) and had higher BMI (mean 32 vs 29 kg/m^2^), hsCRP (3.7 vs 2.4 mg/l), and IL6 (2.7 vs 1.9 pg/ml) levels. (Additional file 1: Table S1).

### Incidence of CVD event and mortality outcome

Among 1671 participants with nonzero follow-up time, 127 incident events (18 fatal CVD events, 41 other deaths and 68 nonfatal CVD events) were observed over a median follow-up period of 8.3 years (interquartile range: 7.2–9.2; 13,093 person-years of follow-up). The unadjusted and age-, sex- adjusted incidence rates of the primary (CVD events and mortality) and secondary (nonfatal and fatal CVD events) are shown in Table 2. The overall age-, sex- adjusted incidence of the primary outcome was 9.91 (95% CI, 8.19–11.64) per 1000 person-years (PYRS). The corresponding incidence rates for subgroups of participants with 0, 1, and ≥2 CSR scores were 5.31 (3.40–7.22), 10.32 (7.16–13.49) and 17.80 (12.94–22.67) per 1000 PYRS, respectively. A similar pattern of linear increase in event rates with increasing number of CSR score was noted for the secondary outcome (Table 2).

**Table 2:** Adjusted and unadjusted event rates for primary and secondary outcome

Outcome	Group	Person-time (PYRS)	No. Event	Unadjusted rate (95% CI) per 1000 PYRS	Age- & sex- adjusted rate (95% CI) per 1000 PYRS
Mortality + CVD event	Overall	13093	127	9.7 (8.15–11.54)	9.91 (8.19–11.64)
	CSR =0	4900	30	6.12 (4.28–8.76)	5.31 (3.40–7.22)
	CSR =1	3989	41	10.28 (7.57–13.96)	10.32 (7.16–13.49)
	CSR ≥2	4204	56	13.32 (10.25–17.3	17.80 (12.94–22.67)
CVD event	Overall	13093	86	6.57 (5.32–8.11)	6.93 (5.28–8.11)
	CSR =0	4900	22	4.49 (2.96–6.82)	4.00 (2.32–5.67)
	CSR =1	3989	28	7.02 (4.85–10.17)	7.07 (4.45–9.69)
	CSR ≥2	4204	36	8.56 (6.18–11.87)	10.77 (7.11–14.43)

*PYRS* person years, *CI* confidence interval, *CVD* cardiovascular disease, *CSR* cumulative social risk

### Cumulative social risk and incident CVD and mortality outcome

**Fig. 1:**
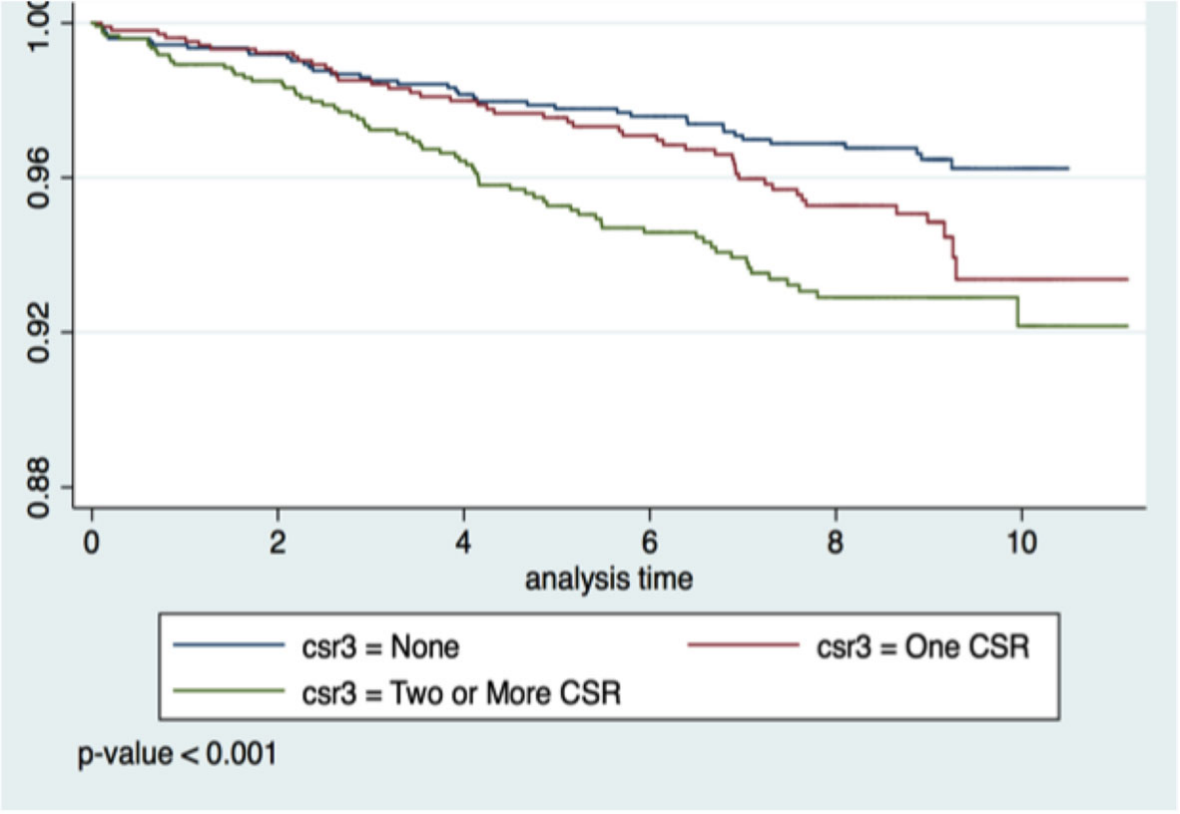
Kaplan Meier even-free survivor curve by Cumulative Social Risk (CSR 0, 1, or ≥2) with adjustment for CVD risk factors*. *Adjusted to mean values of age, sex, smoking status, systolic blood pressure, body mass index, diabetes, and total high-density lipoprotein cholesterol

Figure 1 shows Kaplan Meier event-free survivor curve by CSR (CSR 0, 1, or ≥2) with adjustment for CVD risk factors. Compared to those with a CSR score of 0, individuals with a CSR score of 1 had an age- and sex adjusted HR of 1.94 (95% CI 1.21, 3.11) for incident primary outcomes (composite of CVD events and mortality). This association was only mildly attenuated after adjustment for traditional CVD risk factors (1.85 [95% CI, 1.15–2.97]) (Table 3). The corresponding unadjusted and adjusted HRs for individuals with a CSR score of ≥2 were 3.35 (2.12–5.29) and 2.58 (1.60–4.17), respectively. The association between CSR and incident primary outcomes was attenuated by <5% after additional adjustment for variables that could potentially mediate the relationship, including inflammatory markers (hsCRP, IL6), psychological factors (depression, stress, perceived discrimination), and statin use (Table 3). There was evidence of a graded increase in CVD risk as the CSR score increased from 0 to 1 and ≥2 or more (*p*-value for trend <0.001 for all models) (Table 3).

**Table 3:** Association of cumulative social risk (3 categories) with combined cardiovascular disease and mortality outcome (1671 participants, 127 cases) with adjustment for traditional CVD risk factors and psychosocial factors

Progressive adjustment^a^	CSR = 1, *N* = 501		CSR ≥2, *N* = 573		Per Unit score (trend)	
	HR (95% CI)	*p*-value	HR (95% CI)	*p*-value	HR (95% CI)	*p*-value
Crude	1.68(1.05,2.68)	0.03	2.17(1.39,3.38)	<0.001	1.45(1.17,1.80)	<0.001
Age & sex	1.94(1.21,3.11)	0.01	3.35(2.12,5.29)	<0.001	1.82(1.45,2.28)	<0.001
Above + smoking	1.94(1.21,3.12)	0.01	3.00(1.89,4.76)	<0.001	1.72(1.37,2.15)	<0.001
Above + SBP	1.90(1.19,3.05)	0.01	2.70(1.69,4.31)	<0.001	1.63(1.30,2.04)	<0.001
Above + diabetes	1.83(1.14,2.95)	0.01	2.47(1.54,3.96)	<0.001	1.55(1.24,1.95)	<0.001
Above + BMI	1.84(1.15,2.96)	0.01	2.51(1.56,4.04)	<0.001	1.57(1.24,1.98)	<0.001
Above + TC	1.86(1.16,2.98)	0.01	2.50(1.55,4.03)	<0.001	1.56(1.24,1.97)	<0.001
Above + HDL-c	1.85(1.15,2.97)	0.01	2.58(1.60,4.17)	<0.001	1.59(1.26,2.01)	<0.001
Above + HsCRP	1.85(1.15,2.98)	0.01	2.56(1.59,4.14)	<0.001	1.59(1.26,2.00)	<0.001
Above + IL6	1.82(1.13,2.92)	0.01	2.50(1.55,4.05)	<0.001	1.57(1.24,1.98)	<0.001
Above + CESD	1.80(1.12,2.90)	0.01	2.42(1.49,3.93)	<0.001	1.54(1.22,1.95)	<0.001
Above + Cohen	1.80(1.12,2.89)	0.02	2.41(1.48,3.91)	<0.001	1.54(1.21,1.95)	<0.001
Above + DIS	1.81(1.12,2.93)	0.02	2.43(1.48,4.00)	<0.001	1.54(1.21,1.97)	<0.001
Above + statin	1.81(1.12,2.93)	0.02	2.48(1.51,4.08)	<0.001	1.56(1.22,1.99)	<0.001

^a^The first model is a univariate model; the second model is adjusted for age and sex; each of the subsequent models is constructed by adding the variable listed in corresponding line plus all the variables found in the model in the line above it

*CSR* cumulative risk score, *SBP* systolic blood pressure, *BMI* body mass index, *TC* total cholesterol, *HDL-c* high-density lipoprotein cholesterol

*HsCRP* high-sensitivity C-reactive protein, *IL6* interleukin-6, *CESD* Center for Epidemiologic Studies Depression Scale, Cohen stress scale, *DIS* discrimination score

NB. The *p*-value is a test of statistically significant association between CSR and the clinical outcome. *P*-value for trend (last column) indicates if the association of CSR with clinical outcome, per unit changes in CSR scores, is statistically significant. A *p*-value <0.05 is considered statistically significant

For the secondary outcome (i.e., fatal and nonfatal CVD events), individuals with a CSR score of 1 had an age- and sex adjusted hazard ratio of 1.74 (95% CI 0.99–3.05), which was borderline statistically significant (*p*-value 0.05). (Table 4) This association was attenuated after adjustment for traditional CVD risk factors (1.66 [95% CI 0.95–2.91, *p*-value 0.08]) The corresponding hazard ratios for individuals with a CSR score of ≥2 were 2.60 (1.50–4.49) and 2.12 (1.19–3.76), respectively. The association was only modestly attenuated upon further adjustment for potential mediators described above. There was evidence of a graded increase in CVD risk as the CSR score increased from 0 to 1 and ≥2 or more (*p*-value for trend <0.05) (Table 4).

**Table 4:** Association of cumulative social risk (3 categories) with combined fatal and nonfatal cardiovascular disease outcome (1671 participants and 86 cases) with adjustment for traditional CVD risk factors and psychosocial factors

Progressive adjustment^a^	CSR = 1, *N* = 501		CSR ≥2, *N* = 573		Per Unit score (trend)	
	HR (95% CI)	*p*-value	HR (95% CI)	*p*-value	HR (95% CI)	*p*-value
Crude	1.54(0.88,2.70)	0.13	1.85(1.09,3.15)	0.02	1.35(1.04,1.74)	0.02
Age & sex	1.74(0.99,3.05)	0.05	2.60(1.50,4.49)	<0.001	1.60(1.23,2.10)	<0.001
Above + smoking	1.74(0.99,3.04)	0.05	2.44(1.41,4.24)	<0.001	1.55(1.18,2.03)	<0.001
Above + SBP	1.71(0.97,2.99)	0.06	2.18(1.25,3.82)	0.01	1.47(1.12,1.92)	0.01
Above + diabetes	1.65(0.94,2.89)	0.08	1.99(1.13,3.49)	0.02	1.40(1.06,1.84)	0.02
Above + BMI	1.66(0.95,2.91)	0.08	2.03(1.14,3.59)	0.02	1.41(1.07,1.87)	0.02
Above + TC	1.67(0.95,2.93)	0.07	2.02(1.14,3.57)	0.02	1.41(1.07,1.86)	0.02
Above + HDL-c	1.66(0.95,2.91)	0.08	2.12(1.19,3.76)	0.01	1.45(1.09,1.92)	0.01
Above + HsCRP	1.67(0.95,2.93)	0.07	2.10(1.18,3.74)	0.01	1.44(1.09,1.91)	0.01
Above + IL6	1.66(0.95,2.92)	0.08	2.08(1.17,3.72)	0.01	1.43(1.08,1.90)	0.01
Above + CESD	1.65(0.94,2.90)	0.08	2.04(1.14,3.65)	0.02	1.42(1.07,1.88)	0.02
Above + Cohen	1.62(0.92,2.85)	0.09	2.00(1.12,3.58)	0.02	1.41(1.06,1.87)	0.02
Above + DIS	1.59(0.89,2.82)	0.12	1.92(1.05,3.50)	0.03	1.38(1.03,1.85)	0.03
Above + statin	1.59(0.89,2.82)	0.11	1.96(1.07,3.57)	0.03	1.39(1.04,1.87)	0.03

^a^The first model is a univariate model; the second model is adjusted for age and sex; each of the subsequent models is constructed by adding the variable listed in corresponding line plus all the variables found in the model in the line above it.

*CSR* cumulative risk score, *SBP* systolic blood pressure, *BMI* body mass index, *TC* total cholesterol, *HDL-c* high-density lipoprotein cholesterol

*HsCRP* high-sensitivity C-reactive protein, *IL6* interleukin-6, *CESD* Center for Epidemiologic Studies Depression Scale, Cohen stress scale, *DIS* discrimination score

NB. The *p*-value is a test of statistically significant association between CSR and the clinical outcome. *P*-value for trend (last column) indicates if the association of CSR with clinical outcome, per unit changes in CSR scores, is statistically significant. A *p*-value <0.05 is considered statistically significant.

In a subset of participants with available information on fruit and vegetable consumption, and physical activity (1569 individuals) the association of CSR with the primary or secondary outcome were similar (albeit weaker). (Tables 5 and 6). The associations were only modestly attenuated by adjustment for ideal fruit and vegetable consumption and ideal physical activity (Tables 5 and 6).

**Table 5:** Association of cumulative social risk (3 categories) with combined cardiovascular disease and mortality outcome (1569 participants, 134 cases) with adjustment for traditional CVD risk factors, plus ideal fruit and vegetable consumption, and physical activity

Progressive adjustment	CSR = 1, *N* = 478		CSR ≥2, *N* = 576		Per Unit score (trend)	
	HR (95% CI)	*p*-value	HR (95% CI)	*p*-value	HR (95% CI)	*p*-value
Crude	1.35(0.86,2.10)	0.19	1.75(1.16,2.64)	0.01	1.32(1.08,1.62)	0.01
Age & sex	1.51(0.97,2.37)	0.07	2.55(1.67,3.91)	<0.001	1.60(1.29,1.98)	<0.001
Traditional CV factors	1.42(0.91,2.23)	0.12	1.92(1.22,3.01)	<0.001	1.38(1.11,1.73)	<0.001
Above + ideal diet^a^	1.38(0.88,2.17)	0.16	1.85(1.17,2.91)	0.01	1.36(1.08,1.70)	0.01
Above + ideal activity^b^	1.39(0.88,2.18)	0.16	1.85(1.17,2.91)	0.01	1.36(1.08,1.70)	0.01

*CSR* cumulative risk score, *CV* cardiovascular, Ideal diet – refers to ideal consumption of fruit and vegetables; Ideal activity – refers to ideal physical activity according to AHA definition of Life’s Simple 7

Traditional CV factors - adjusted for age, sex, smoking, systolic blood pressure, diabetes, body mass index, total cholesterol and high-density lipoprotein cholesterol

^a^This model is adjusted for traditional CV risk factors above plus ideal fruit and vegetable consumption

^b^This model is adjusted for traditional CV risk factors above, and ideal fruit and vegetable consumption, plus ideal physical activity

NB. The *p*-value is a test of statistically significant association between CSR and the clinical outcome. *P*-value for trend (last column) indicates if the association of CSR with clinical outcome, per unit changes in CSR scores, is statistically significant. A *p*-value <0.05 is considered statistically significant

**Table 6:** Association of cumulative social risk (3 categories) with combined fatal and nonfatal cardiovascular disease outcome (1569 participants, 92 cases) with adjustment for traditional CVD risk factors, plus ideal fruit and vegetable consumption, and physical activity

Progressive adjustment	CSR = 1, *N* = 478		CSR ≥2, *N* = 576		Per Unit score (trend)	
	HR (95% CI)	*p*-value	HR (95% CI)	*p*-value	HR (95% CI)	*p*-value
Crude	1.34(0.78,2.28)	0.29	1.63(0.99,2.69)	0.05	1.27(1.00,1.63)	0.05
Age & sex	1.48(0.87,2.53)	0.15	2.27(1.36,3.79)	<0.001	1.51(1.17,1.95)	<0.001
Traditional CV	1.42(0.83,2.42)	0.2	1.81(1.05,3.12)	0.03	1.34(1.03,1.76)	0.03
Above + ideal diet^a^	1.36(0.79,2.32)	0.27	1.71(0.99,2.95)	0.05	1.31(1.00,1.71)	0.05
Above + ideal activity^b^	1.35(0.79,2.31)	0.27	1.70(0.99,2.94)	0.06	1.30(0.99,1.71)	0.06

*CSR* cumulative risk score, Ideal diet – refers to ideal consumption of fruit and vegetables; Ideal activity – refers to ideal physical activity according to AHA definition of Life’s Simple 7

Traditional CV factors - adjusted for age, sex, smoking, systolic blood pressure, diabetes, body mass index, total cholesterol and high-density lipoprotein cholesterol

^a^This model is adjusted for traditional CV risk factors above plus ideal fruit and vegetable consumption

^b^This model is adjusted for traditional CV risk factors above, and ideal fruit and vegetable consumption, plus ideal physical activity

NB. The *p*-value is a test of statistically significant association between CSR and the clinical outcome. *P*-value for trend (last column) indicates if the association of CSR with clinical outcome, per unit changes in CSR scores, is statistically significant. A *p*-value <0.05 is considered statistically significant

### Cumulative social risk and carotid artery intima-media thickness

In cross sectional analyses of the association between CSR and presence of significant CIMT (defined as CIMT >1 mm), individuals with a CSR score of 1 had an odds ratio of 1.31 (0.83–2.07) compared to those with a CSR score of 0 in a model adjusted for age, sex and traditional CVD risk factors. The corresponding odds ratio for individuals with CSR score of ≥2 was 1.86 (1.15–3.02). Thus, there was evidence of a graded increase in risk as the CSR score increased from 0 to 1 and ≥2 (odds ratio = 1.36; 1.07–1.73 in adjusted model; *p*-value for trend = 0.001) (Table 7).

**Table 7:** Association of cumulative social risk (3 categories) with significant CIMT (CIMT >1 mm, 689 participants)

Progressive adjustment^a^	CSR = 1, *N* = 209		CSR ≥2, *N* = 190		Per Unit score (trend)	
	OR (95% CI)	*p*-value	OR (95% CI)	*p*-value	OR (95% CI)	*p*-value
Crude	1.16(0.76,1.77)	0.5	1.46(0.96,2.23)	0.08	1.21(0.98,1.49)	0.08
Age & sex	1.37(0.88,2.14)	0.16	2.15(1.36,3.40)	<0.001	1.46(1.16,1.84)	<0.001
Above + smoking	1.37(0.88,2.13)	0.16	2.15(1.36,3.39)	<0.001	1.46(1.16,1.84)	<0.001
Above + SBP	1.31(0.84,2.05)	0.24	1.86(1.16,2.97)	0.01	1.36(1.08,1.72)	0.01
Above + diabetes	1.32(0.84,2.07)	0.23	1.87(1.17,3.00)	0.01	1.37(1.08,1.73)	0.01
Above + BMI	1.30(0.83,2.04)	0.26	1.79(1.11,2.89)	0.02	1.34(1.05,1.70)	0.02
Above + TC	1.31(0.83,2.07)	0.24	1.86(1.15,3.01)	0.01	1.36(1.07,1.73)	0.01
Above + HDL-c	1.31(0.83,2.07)	0.25	1.86(1.15,3.02)	0.01	1.36(1.07,1.73)	0.01
Above + HsCRP	1.31(0.83,2.07)	0.24	1.86(1.15,3.02)	0.01	1.36(1.07,1.73)	0.01
Above + IL6	1.31(0.83,2.07)	0.25	1.86(1.15,3.02)	0.01	1.36(1.07,1.74)	0.01
Above + CESD	1.30(0.82,2.05)	0.27	1.81(1.11,2.95)	0.02	1.34(1.05,1.71)	0.02
Above + Cohen	1.30(0.82,2.06)	0.27	1.81(1.11,2.95)	0.02	1.34(1.05,1.72)	0.02
Above + DIS	1.35(0.84,2.14)	0.21	1.89(1.14,3.13)	0.01	1.37(1.07,1.77)	0.01
Above + statin	1.34(0.84,2.14)	0.22	1.88(1.13,3.13)	0.01	1.37(1.06,1.77)	0.01

^a^The first model is a univariate model; the second model is adjusted for age and sex; each of the subsequent models is constructed by adding the variable listed in corresponding line plus all the variables found in the model in the line above it

*CSR* cumulative risk score, *SBP* systolic blood pressure, *BMI* body mass index, *TC* total cholesterol, *HDL-c* high-density lipoprotein cholesterol

*HsCRP* high-sensitivity C-reactive protein, *IL6* interleukin-6, *CESD* Center for Epidemiologic Studies Depression Scale, Cohen stress scale, *DIS* discrimination score

NB. The *p*-value is a test of statistically significant association between CSR and the clinical outcome. *P*-value for trend (last column) indicates if the association of CSR with clinical outcome, per unit changes in CSR scores, is statistically significant. A *p*-value <0.05 is considered statistically significant

### Subsidiary analyses

To examine any effect of categorization of CSR score used in the main analyses (i.e., 0, 1 and ≥2, we performed a sensitivity analyses by categorizing CSR scores into 4 categories (0, 1, 2, ≥3), which yielded broadly similar results (Additional file 1: Tables S2 and S3).

## Discussion

Using prospective data from a cohort composed of comparable proportions of Black and White participants followed for an average of 8.3 years, we demonstrated that accumulation of four social risk factors (belonging to racial minority group, low social income, low education, and single living status) is associated in a gradient manner with increased risk of combined all-cause mortality and CVD events, as well as with presence of significant CIMT, which is a marker of subclinical atherosclerosis. Cumulative social risk was associated with several traditional CVD risk factors including smoking, blood pressure, diabetes, BMI, and family history of coronary artery disease, as well as with high levels of inflammatory marker (hsCRP, IL6) and adverse psychosocial factors (depression, stress, perceived discrimination). Adjustment for traditional CVD risk factors, as well as for inflammatory and psychosocial factors, several of which may be in the causal pathway of the association, did not materially attenuate the relative risks for the primary outcome of mortality or non-fatal CVD events. These findings suggest a strong independent effect of CSR on risk of mortality and CVD.

Previous studies have shown that the presence of single measures of socioeconomic disadvantage, such as ethnic minority status, low education or low income, is associated with adverse cardiovascular outcomes [3, 6, 7, 22–24]. It has also been demonstrated that these different measures are not necessarily interchangeable [25]. Furthermore, studies have also shown that the duration of time that individuals spent living in a disadvantaged socioeconomic position increases the risk of adverse CVD outcomes [8, 26]. However, few studies have evaluated the cumulative effect of the simultaneous presence of multiple social risk factors on the risk of non-fatal CVD outcomes and mortality and the construct of cumulative social risk has been defined differently in studies [7, 27]. Our findings complement and supplement findings from previous studies by using a 4-variable construct of CSR, assessing its association with several traditional and novel CVD risk factors, as well as its associations with risk of non-fatal CVD events and all-cause mortality. For instance, while extant studies have only investigated mortality [7], we also investigated incident CVD events and subclinical disease, thus providing a more robust and comprehensive assessment of the relation of CSR to CVD.

The associations between CSR and several traditional CVD risk factors, inflammatory factors, depression, perceived discrimination, and stress suggest that these factors may potentially mediate or confound the primary association between CSR and CVD and mortality outcomes. In particular, there is a growing evidence supporting the role of psychosocial factors such as perceived discrimination, stress and depression in mediating and modifying the relationship between disadvantageous social exposures (such as ethnic minority status and low income) and clinical outcomes [9–11]. That the association was minimally attenuated after adjustment for the factors discussed above, may suggest a strong independent effect of CSR. Important related considerations, however, include measurement error in mediators and confounders, as well as unidentified effect modifications and interactions.

We also investigated the effect of adjustment for ideal fruit and vegetable consumption in attempt to partially account for more proximal potential effect-mediating social constructs, which did not materially attenuate the association. These proximal social constructs which include, but are not limited to, factors such as health behavior (e.g., medical compliance, healthy eating index, physical activity) and access to health care (e.g., having medical insurance and primary care follow-up) are not likely to be fully captured by the limited adjustment that was possible in the current analyses. Behavioral risk factors are of importance, as it has been hypothesized that social stressors adversely affect health behaviors, such as diet, physical activity, smoking habit, and alcohol use, partly accounting for the high rates of obesity and CVD seen among minorities and lower social status populations who may experience a greater number of stressors than non-minority populations.

Epigenetic modifications, i.e., heritable and potentially modifiable markers that regulate gene expression without changing the underlying DNA sequence, provide potential mechanistic explanation for social determinants of CVD. Epigenetic markers are responsive to non-biological and environmental exposures, particularly those encountered in early life [28]. Indeed, existing evidence suggests an association between socioeconomic circumstances and global and genome-wide DNA methylation [29–32]. Hence, further studies should investigate the interrelation of social and biological risk factors and CVD outcomes.

The contribution of access to health care to racial disparities observed among the US general population was demonstrated by a recent study of a large cohort of US veterans, which showed that African-American veterans, in contrast to non-veteran African-Americans, do not have an increased risk of CVD and mortality outcomes [33]. On the other hand, a nationwide study of the association between income and life expectancy in the US using deidentified tax data and Social Security Administration death records found that differences in life expectancy between geographic locations for individuals in the lowest income quartile were correlated to health behavior factors such smoking rate and exercise rate, and local area characteristics such as fraction of college graduates, fraction of immigrants, and government expenditures. This important study, which found a life expectancy difference of 14.8 years for men and 10.1 years for women between the richest 1% and the poorest 1% individuals, highlighted the intricate nature of wealth-health relationship [34].

Our findings indicate the need to take into account a construct for defining social disadvantage in CVD prevention and control strategies. The social environment should be considered as important target for reducing the risk and burden of CVD. Socially disadvantaged individuals may require more attention in addressing the risk of CVD and adverse clinical outcomes. The inclusion of a CSR index in CVD risk assessment tools may help improve the identification of at-risk individuals for targeting with interventions. For instance, in the UK, social deprivation has been included in CVD risk assessment tools, resulting in improvement in risk prediction [35]. Also, in analyses combining data from the Atherosclerosis Risk in Communities (ARIC) Study and NHANES, improvements in calibration of CVD risk prediction models were noted after including data on social risk factors [36]. Evidence indicates that not accounting for social factors may lead to inaccurate CVD risk estimation. For example, the Framingham risk score overestimates the risk of coronary heart disease in high-socioeconomic status individuals and underestimates the risk in low–socioeconomic status individuals. Thus, recent studies have begun to evaluate the potential benefit of including socioeconomic status in risk prediction models [37]. Accounting for the totality of social disadvantage may therefore improve the prediction of CVD risk.

Our findings also have policy implications. Given the multidimensional and intricate nature of social risk factors, multi-lateral collaboration among various sectors to address root causes of social disadvantage such as lack of education, unsafe neighborhood, and unhealthy behavior is needed. For instance, a multi-pronged approach involving governmental and nongovernmental organizations and communities to target early childhood education and foster supportive community environments can help to shift life-course trajectories, equipping children with tools to break the cycle of illiteracy and poverty. Such interventions promote health and would be cost saving in the long-term.

Our study has a number of limitations. First, because of modest statistical power, we merged categories of CSR in order to avoid low prevalence groups and provide for more precise estimates of effect. Second, we were unable to investigate clinical outcomes (e.g., mortality, coronary disease, stroke) separately. Third, we did not assess potential up-stream effect mediators that may be associated with CSR, such as health-access, health-care seeking behavior and medical compliance. Fourth, the definition of CSR can vary between studies and the current CSR construct may not be applicable in other settings. In addition, it is not clear if the various components of CSR carry equal weight. Therefore, further studies are needed to assess the generalizability of these findings. Nevertheless, the strengths of our study include an examination of a range of social risk factors, and their cumulative deleterious effects in a cohort composed of Black and White participants, accounting for various aspects of social disadvantage, as well as their combined effect on subclinical cardiovascular disease, which has not been extensively examined previously.

## Conclusion

In summary, we found that an accumulation of social risk factors increased the likelihood of CVD events and deaths in a cohort composed of comparable proportions of Whites and Blacks; we also found a graded association between CSR and presence of significant CIMT in cross-sectional analyses. The sizable toll that simultaneous exposure to a multitude of social risks factors take on CVD risk points toward the need for appropriate clinical and preventive strategies that allocate sufficient and specific resources to address the needs of socially disadvantaged groups.

## Additional file

Additional file 1: Table S1. Study characteristics by race. **Table S2** Study characteristic by cumulative social risk (4 categories). **Table S3** Association of cumulative social risk (4 categories) with combined cardiovascular disease and mortality outcome (1671 participants, 127 cases). (DOCX 120 kb)

## Abbreviations

BMI: Body mass index
CES-D: Center for epidemiologic studies depression scale
CIMT: Carotid artery intima-media thickness
CSR: Cumulative social risk
CVD: Cardiovascular disease
ELISA: Enzyme linked immunosorbent assay
HDL-c: High-density lipoprotein cholesterol
HR: Hazard ratio
hsCRP: High sensitivity C-reactive protein
IL6: Interleukin-6
LS7: Life’s simple 7

## References

[1] Stewart JA, Dundas R, Howard RS, Rudd AG, Wolfe CD (1999). Ethnic differences in incidence of stroke: prospective study with stroke register. BMJ.

[2] Lynch GF, Gorelick PB (2000). Stroke in African Americans. Neurol Clin.

[3] Mensah GA, Mokdad AH, Ford ES, Greenlund KJ, Croft JB (2005). State of disparities in cardiovascular health in the United States. Circulation.

[4] Thom T, Haase N, Rosamond W, Howard VJ, Rumsfeld J, Manolio T, Zheng ZJ, Flegal K, O'Donnell C, Kittner S, Lloyd-Jones D, Goff DC, Jr., Hong Y, Adams R, Friday G, Furie K, Gorelick P, Kissela B, Marler J, Meigs J, Roger V, Sidney S, Sorlie P, Steinberger J, Wasserthiel-Smoller S, Wilson M, Wolf P, American Heart Association Statistics C and Stroke Statistics S. Heart disease and stroke statistics--2006 update: a report from the American Heart Association Statistics Committee and Stroke Statistics Subcommittee. Circulation. 2006;113:e85–151. Accessed 16 Nov 2016. doi:10.1161/CIRCULATIONAHA.105.171600. 10.1161/CIRCULATIONAHA.105.171600 16407573

[5] Murray CJ, Kulkarni SC, Michaud C, Tomijima N, Bulzacchelli MT, Iandiorio TJ, Ezzati M. Eight Americas: investigating mortality disparities across races, counties, and race-counties in the United States. PLoS Med 2006;3:e260. Accessed 16 Nov 2016. doi:10.1371/journal.pmed.0030260. 10.1371/journal.pmed.0030260 PMC1564165 16968116

[6] Erqou S, Kip KE, Mulukutla SR, Aiyer AN, Reis SE (2016). Endothelial dysfunction and racial disparities in mortality and Adverse cardiovascular disease outcomes. Clin Cardiol.

[7] Caleyachetty R, Echouffo-Tcheugui JB, Shimbo D, Zhu W, Muennig P (2014). Cumulative social risk and risk of death from cardiovascular diseases and all-causes. Int J Cardiol.

[8] Pollitt RA, Rose KM, Kaufman JS (2005). Evaluating the evidence for models of life course socioeconomic factors and cardiovascular outcomes: a systematic review. BMC Public Health.

[9] Assari S (2016). Race and ethnic differences in additive and multiplicative effects of depression and anxiety on cardiovascular risk. Int J Prev Med.

[10] Brewer LC, Cooper LA (2014). Race, discrimination, and cardiovascular disease. Virtual Mentor.

[11] Parashar S, Rumsfeld JS, Reid KJ, Buchanan D, Dawood N, Khizer S, Lichtman J, Vaccarino V, Investigators PR (2009). Impact of depression on sex differences in outcome after myocardial infarction. Circ Cardiovasc Qual Outcomes.

[12] Mulukutla SR, Venkitachalam L, Bambs C, Kip KE, Aiyer A, Marroquin OC, Reis SE (2010). Black race is associated with digital artery endothelial dysfunction: results from the heart SCORE study. Eur Heart J.

[13] Aiyer AN, Kip KE, Marroquin OC, Mulukutla SR, Edmundowicz D, Reis SE (2007). Racial differences in coronary artery calcification are not attributed to differences in lipoprotein particle sizes: the heart strategies concentrating on risk evaluation (heart SCORE) study. Am Heart J.

[14] Erqou S, Kip KE, Mulukutla SR, Aiyer AN, Reis SE (2015). Racial differences in the burden of coronary artery calcium and carotid intima media thickness between blacks and Whites. Neth Heart J.

[15] Radloff LS (1977). The CES-D scale: a self-reported depression scale for research in the general population. Appl Psychol Meas.

[16] Cohen S, Kamarck T, Mermelstein R (1983). A global measure of perceived stress. J Health Soc Behav.

[17] Krieger N, Smith K, Naishadham D, Hartman C, Barbeau EM (2005). Experiences of discrimination: validity and reliability of a self-report measure for population health research on racism and health. Soc Sci Med.

[18] Ainsworth BE, Jacobs DR, Leon AS (1993). Validity and reliability of self-reported physical activity status: the lipid research clinics questionnaire. Med Sci Sports Exerc.

[19] Lloyd-Jones DM, Hong Y, Labarthe D, Mozaffarian D, Appel LJ, Van Horn L, Greenlund K, Daniels S, Nichol G, Tomaselli GF, Arnett DK, Fonarow GC, Ho PM, Lauer MS, Masoudi FA, Robertson RM, Roger V, Schwamm LH, Sorlie P, Yancy CW, Rosamond WD, American Heart Association Strategic Planning Task F and Statistics C (2010). Defining and setting national goals for cardiovascular health promotion and disease reduction: the American Heart Association's strategic Impact Goal through 2020 and beyond. Circulation.

[20] Bambs C, Kip KE, Dinga A, Mulukutla SR, Aiyer AN, Reis SE (2011). Low prevalence of "ideal cardiovascular health" in a community-based population: the heart strategies concentrating on risk evaluation (heart SCORE) study. Circulation.

[21] Rifas-Shiman SL, Willett WC, Lobb R, Kotch J, Dart C, Gillman MW (2001). PrimeScreen, a brief dietary screening tool: reproducibility and comparability with both a longer food frequency questionnaire and biomarkers. Public Health Nutr.

[22] Goyal A, Bhatt DL, Steg PG, Gersh BJ, Alberts MJ, Ohman EM, Corbalan R, Eagle KA, Gaxiola E, Gao R, Goto S, D'Agostino RB, Califf RM, Smith SC, Wilson PW, Reduction of Atherothrombosis for Continued Health Registry I (2010). Attained educational level and incident atherothrombotic events in low- and middle-income compared with high-income countries. Circulation.

[23] Bucholz EM, Rathore SS, Gosch K, Schoenfeld A, Jones PG, Buchanan DM, Spertus JA, Krumholz HM (2011). Effect of living alone on patient outcomes after hospitalization for acute myocardial infarction. Am J Cardiol.

[24] Udell JA, Steg PG, Scirica BM, Smith SC, Ohman EM, Eagle KA, Goto S, Cho JI, Bhatt DL, Investigators REoAfCHR (2012). Living alone and cardiovascular risk in outpatients at risk of or with atherothrombosis. Arch Intern Med.

[25] Daly MC, Duncan GJ, McDonough P, Williams DR (2002). Optimal indicators of socioeconomic status for health research. Am J Public Health.

[26] Ljung R, Hallqvist J (2006). Accumulation of adverse socioeconomic position over the entire life course and the risk of myocardial infarction among men and women: results from the Stockholm heart epidemiology program (SHEEP). J Epidemiol Community Health.

[27] Lawlor DA, Ebrahim S, Davey SG (2005). Adverse socioeconomic position across the lifecourse increases coronary heart disease risk cumulatively: findings from the British women's heart and health study. J Epidemiol Community Health.

[28] Glier MB, Green TJ, Devlin AM (2014). Methyl nutrients, DNA methylation, and cardiovascular disease. Mol Nutr Food Res.

[29] Borghol N, Suderman M, McArdle W, Racine A, Hallett M, Pembrey M, Hertzman C, Power C, Szyf M (2012). Associations with early-life socio-economic position in adult DNA methylation. Int J Epidemiol.

[30] Subramanyam MA, Diez-Roux AV, Pilsner JR, Villamor E, Donohue KM, Liu Y, Jenny NS. Social factors and leukocyte DNA methylation of repetitive sequences: the multi-ethnic study of atherosclerosis. PLoS One 2013;8:e54018. Accessed 16 Nov 2016. doi:10.1371/journal.pone.0054018. 10.1371/journal.pone.0054018 PMC3539988 23320117

[31] McGuinness D, McGlynn LM, Johnson PC, MacIntyre A, Batty GD, Burns H, Cavanagh J, Deans KA, Ford I, McConnachie A, McGinty A, McLean JS, Millar K, Packard CJ, Sattar NA, Tannahill C, Velupillai YN, Shiels PG (2012). Socio-economic status is associated with epigenetic differences in the pSoBid cohort. Int J Epidemiol.

[32] Stringhini S, Polidoro S, Sacerdote C, Kelly RS, van Veldhoven K, Agnoli C, Grioni S, Tumino R, Giurdanella MC, Panico S, Mattiello A, Palli D, Masala G, Gallo V, Castagne R, Paccaud F, Campanella G, Chadeau-Hyam M, Vineis P (2015). Life-course socioeconomic status and DNA methylation of genes regulating inflammation. Int J Epidemiol.

[33] Kovesdy CP, Norris KC, Boulware LE, Lu JL, Ma JZ, Streja E, Molnar MZ, Kalantar-Zadeh K (2015). Association of race with mortality and cardiovascular events in a large cohort of US veterans. Circulation.

[34] Hippisley-Cox J, Coupland C, Vinogradova Y, Robson J, Minhas R, Sheikh A, Brindle P (2008). Predicting cardiovascular risk in England and Wales: prospective derivation and validation of QRISK2. BMJ.

[35] Chetty R, Stepner M, Abraham S, Lin S, Scuderi B, Turner N, Bergeron A, Cutler D (2016). The association between income and life expectancy in the United States, 2001-2014. JAMA.

[36] Fiscella K, Tancredi D, Franks P (2009). Adding socioeconomic status to Framingham scoring to reduce disparities in coronary risk assessment. Am Heart J.

[37] Brindle PM, McConnachie A, Upton MN, Hart CL, Davey Smith G, Watt GC (2005). The accuracy of the Framingham risk-score in different socioeconomic groups: a prospective study. Br J Gen Pract.

