# Supplementary material for: Association of cumulative social risk with mortality and adverse cardiovascular disease outcomes
Source: BMC Cardiovasc Disord. 2017 May 8;17:110. doi: 10.1186/s12872-017-0539-9 (PMC5422978; doi:10.1186/s12872-017-0539-9)
Supplement: Additional file 1: Table S1. — Study characteristics by race. Table S2 Study characteristic by cumulative social risk (4 categories). Table S3 Association of cumulative social risk (4 categories) with combined cardiovascular disease and mortality outcome (1671 participants, 127 cases). (DOCX 120 kb) [file 12872_2017_539_MOESM1_ESM.docx]

Supplementary table 1: Study characteristics by race

| ***Traditional***  ***risk factors*** | White (N=971) | Black (N= 715) | p-value |
| --- | --- | --- | --- |
|  | Mean (SD) or N (%) | Mean (SD) or N (%) |  |
| Age (years) | 60 (7) | 58 (7) | <0.001 |
| Male | 360 (37%) | 212 (30%) | 0.001 |
| Current smoker | 81 (8%) | 101 (14%) | <0.001 |
| Systolic BP | 133 (18) | 141 (20) | <0.001 |
| Diastolic BP | 79 (10) | 83 (10) | <0.001 |
| Hx of diabetes | 50 (5%) | 115 (16%) | <0.001 |
| Hx hypertension | 297 (31%) | 403 (56%) | <0.001 |
| BMI (kg/m2) | 29 (5) | 32 (6) | <0.001 |
| TC (mg/dL) | 216 (41) | 209 (45) | 0.001 |
| HDL-c (mg/dL) | 57 (15) | 58 (14) | 0.21 |
| TG (mg/dL) | 132 (80) | 110 (63) | <0.001 |
| ***Components of CSR*** |  |  |  |
|  |  |  |  |
| Low income | 183 (19%) | 300 (42%) | <0.001 |
| Single living | 275 (28%) | 431 (60%) | <0.001 |
| < High school | 15 (2%) | 21 (3%) | 0.051 |
| ***Other variables*** |  |  |  |
|  |  |  |  |
| Statin use | 212 (60%) | 137 (39%) | 0.20 |
| Family hx of CAD | 331 (34%) | 283 (40%) | 0.021 |
| Depression* | 94 (10%) | 106 (15%) | 0.001 |
| CESD score | 6.4 (7.4) | 7.6 (8.7) | 0.003 |
| Cohen stress score | 4.11 (2.92) | 4.49 (3.15) | 0.01 |
| DIS score | 9.9 (6.1) | 13 (6) | <0.001 |
| Serum creatinine | 0.90 (0.26) | 0.95 (0.28) | <0.001 |
| HsCRP (mg/l) | 2.36 (4.14) | 3.74 (5.92) | <0.001 |
| IL-6 (pg/ml) | 1.87 (1.48) | 2.69 (2.50) | <0.001 |

Hx – history, BP - blood pressure, BMI – body mass index, < High school – did not complete high school, TC – total cholesterol, HDL-c – high-density lipoprotein cholesterol, TG – triglycerides, HsCRP – high-sensitivity C-reactive protein, IL6 - interleukin-6, CESD – Center for Epidemiologic Studies Depression Scale, Cohen stress scale, DIS – discrimination score. *Depression was defined as CES-D score ≥16. NB. The p-value is a test for statistical significant difference in the distribution of the respective variables between Blacks and Whites. A p-value <0.05 is considered statistically significant.

Supplementary table 2: Study characteristic by cumulative social risk (4 categories)

| ***Traditional***  ***risk factors*** | Overall  N = 1731 | CSR= 0  N = 607 | CSR = 1  N = 520 | CSR = 2  N = 370 | CSR >= 3  N = 234 | p-value |
| --- | --- | --- | --- | --- | --- | --- |
| Age (yrs) | 59.1 (7.4) | 59.7 (7.3) | 59.2 (7.6) | 58.1 (7.3) | 58.8 (7.5) | 0.006 |
| Male | 593 (34.3) | 270 (44.5%) | 185 (35.6%) | 79 (21.4%) | 59 (25.2%) | <0.001 |
| Smoker | 188 (10.9) | 43 (7.1%) | 44 (8.5%) | 51 (13.9%) | 50 (21.4%) | <0.001 |
| SBP (mmHg) | 136.4 (19.4) | 133.1 (18.1) | 134.9 (18.2) | 138.6(20.5) | 144.5 (21.1) | <0.001 |
| DBP (mmHg) | 80.7 (10.4) | 79.0 (10.0) | 80.4 (9.8) | 81.6 (10.6) | 84.2 (11.2) | <0.001 |
| Diabetes | 169 (9.8) | 31 (5.11%) | 43 (8.27%) | 46 (12.43%) | 49 (20.94%) | <0.001 |
| Hypertension | 727 (42.0) | 190 (31.3%) | 205 (39.4%) | 190 (51.4%) | 142 (60.7%) | <0.001 |
| BMI (kg/m2) | 30.0 (6.1) | 28.34 (5.27) | 29.82 (5.79) | 30.88 (6.02) | 33.04 (7.60) | <0.001 |
| TC (mg/dL) | 213.23(42.6) | 214.3(41.4) | 213.6 (41.3) | 214.8 (43.3) | 207.3(47.1) | 0.14 |
| HDL-c (mg/dL) | 57.6 (14.8) | 56.8(15.2) | 57.1 (14.2) | 59.5 (15.0) | 57.9 (14.6) | 0.033 |
| TG (mg/dL) | 122.6 (73.3) | 130.8 (74.9) | 122.6 (70.9) | 113.0 (79.8) | 116.3 (60.4) | 0.001 |
| ***Components of CSR*** |  |  |  |  |  |  |
|  |  |  |  |  |  |  |
| Race-Black | 715 (41.3%) | 0 (0.00%) | 208 (40.0%) | 275 (74.3%) | 232 (99.2%) | <0.001 |
| Low income | 582 (29.1%) | 0 (0.00%) | 111 (18.7%) | 195 (44.2%) | 276 (98.9%) | <0.001 |
| Single living | 849 (42.5%) | 0 (0.00%) | 223 (37.6%) | 350 (79.2%) | 276 (98.9%) | <0.001 |
| < High school | 47 (2.4%) | 0 (0.00%) | 12 (2.0%) | 8 (1.8%) | 27 (9.7%) | <0.001 |
| ***Other variables*** |  |  |  |  |  |  |
|  |  |  |  |  |  |  |
| Statin use | 361 (20.9%) | 141 (23.2%) | 108 (20.8%) | 66 (17.9%) | 46 (19.7%) | 0.24 |
| Family hx CAD | 628 (36.3%) | 194 (31.7%) | 182 (35.0%) | 148 (40.0%) | 104 (44.4%) | <0.001 |
| Depression* | 204 (11.8%) | 39 (6.4%) | 54 (10.4%) | 61 (16.5%) | 50 (21.4%) | <0.001 |
| CESD score | 6.86 (7.98) | 5.29 (6.26) | 6.50 (7.66) | 8.18 (8.83) | 9.63 (9.96) | <0.001 |
| Cohen score | 4.26 (3.01) | 3.71 (2.68) | 4.28 (2.99) | 4.51 (3.25) | 5.21 (3.22) | <0.001 |
| DIS score | 11.23 (6.15) | 9.58 (6.03) | 11.43 (6.10) | 12.58 (5.88) | 12.96 (5.94) | <0.001 |
| Creatinine (mg/dl) | 0.92 (0.27) | 0.91 (0.18) | 0.93 (0.33) | 0.92 (0.21) | 0.95 (0.39) | 0.29 |
| HsCRP (mg/l) | 2.91 (4.98) | 2.34 (4.62) | 2.64 (3.65) | 3.03 (5.31) | 4.83 (7.04) | <0.001 |
| IL6 (pg/ml) | 2.21 (2.02) | 1.78 (1.48) | 2.20 (1.63) | 2.36 (1.78) | 3.15 (3.51) | <0.001 |

CSR – cumulative risk score, SBP - systolic blood pressure, BMI – body mass index, TC – total cholesterol, HDL-c – high-density lipoprotein cholesterol, HsCRP – high-sensitivity C-reactive protein, IL6 - interleukin-6, CESD – Center for Epidemiologic Studies Depression Scale, Cohen stress scale, DIS – discrimination score.

NB. The p-value is a test for statistical significant difference in the distribution of the respective variables across the categories of CSR. A p-value <0.05 is considered statistically significant.

Supplementary table 3: Association of cumulative social risk (4 categories) with combined cardiovascular disease and mortality outcome (1671 participants, 127 cases)

| Progressive adjustment* | CSR = 1, N = 501 | | CSR = 2, N = 354 | | CSR>=3, N = 219 | |
| --- | --- | --- | --- | --- | --- | --- |
|  | HR (95% CI) | p-value | HR (95% CI) | p-value | HR (95% CI) | p-value |
| Crude | 1.68(1.05,2.68) | 0.03 | 2.18(1.35,3.55) | <0.001 | 2.14(1.21,3.76) | 0.01 |
| Age & sex | 1.94(1.21,3.12) | 0.01 | 3.62(2.19,5.98) | <0.001 | 2.96(1.67,5.26) | <0.001 |
| Above + smoking | 1.95(1.21,3.12) | 0.01 | 3.40(2.05,5.62) | <0.001 | 2.49(1.39,4.45) | <0.001 |
| Above + SBP | 1.91(1.19,3.06) | 0.01 | 3.10(1.87,5.15) | <0.001 | 2.18(1.21,3.93) | 0.01 |
| Above + Diabetes | 1.84(1.14,2.95) | 0.01 | 2.84(1.71,4.73) | <0.001 | 1.98(1.09,3.59) | 0.02 |
| Above + BMI | 1.84(1.15,2.96) | 0.01 | 2.87(1.72,4.78) | <0.001 | 2.02(1.10,3.68) | 0.02 |
| Above + TC | 1.86(1.16,2.98) | 0.01 | 2.84(1.71,4.74) | <0.001 | 2.02(1.10,3.68) | 0.02 |
| Above + HDL-c | 1.85(1.15,2.97) | 0.01 | 2.94(1.76,4.91) | <0.001 | 2.08(1.14,3.80) | 0.02 |
| Above + HsCRP | 1.85(1.15,2.98) | 0.01 | 2.93(1.75,4.90) | <0.001 | 2.03(1.11,3.74) | 0.02 |
| Above + Il6 | 1.81(1.13,2.92) | 0.01 | 2.87(1.72,4.80) | <0.001 | 1.95(1.06,3.61) | 0.03 |
| Above + CESD | 1.80(1.12,2.89) | 0.02 | 2.78(1.66,4.66) | <0.001 | 1.89(1.02,3.50) | 0.04 |
| Above + Cohen | 1.79(1.11,2.87) | 0.02 | 2.79(1.67,4.67) | <0.001 | 1.85(0.99,3.44) | 0.05 |
| Above + DIS | 1.80(1.11,2.92) | 0.02 | 2.78(1.63,4.73) | <0.001 | 1.94(1.03,3.63) | 0.04 |
| Above + statin | 1.81(1.12,2.93) | 0.02 | 2.81(1.65,4.78) | <0.001 | 1.98(1.06,3.72) | 0.03 |

*The first model is a univariate model; the second model is adjusted for age and sex; each of the subsequent models is constructed by adding the variable listed in corresponding line plus all the variables found in the model in the line above it.

CSR – cumulative risk score, SBP - systolic blood pressure, BMI – body mass index, TC – total cholesterol, HDL-c – high-density lipoprotein cholesterol

HsCRP – high-sensitivity C-reactive protein, IL6 - interleukin-6, CESD – Center for Epidemiologic Studies Depression Scale, Cohen stress scale, DIS – discrimination score

NB. The p-value is a test of statistically significant association between CSR and the clinical outcome. A p-value <0.05 is considered statistically significant.
